# Intravesical Injection of Botulinum Toxin Type A in Patients with Refractory Overactive Bladder—Results between Young and Elderly Populations, and Factors Associated with Unfavorable Outcomes

**DOI:** 10.3390/toxins15020095

**Published:** 2023-01-19

**Authors:** Yin-Chien Ou, Yao-Lin Kao, Yi-Hui Ho, Kuan-Yu Wu, Hann-Chorng Kuo

**Affiliations:** 1 Department of Urology, National Cheng Kung University Hospital, College of Medicine, National Cheng Kung University, Tainan 704, Taiwan; 2 Department of Anesthesiology, National Cheng Kung University Hospital, College of Medicine, National Cheng Kung University, Tainan 704, Taiwan; 3 Department of Urology, Hualien Tzu Chi Hospital, Buddhist Tzu Chi Medical Foundation and Tzu Chi University, Hualien 970, Taiwan

**Keywords:** botulinum toxins, type A, urinary bladder, overactive, aged, postoperative complications

## Abstract

Intravesical botulinum toxin type A (BoNT-A) injection has been recognized as the standard treatment for refractory overactive bladder (OAB). However, its therapeutic efficacy and safety have not been thoroughly reviewed in elderly patients. This study aims to provide treatment outcomes for patients aged ≥75 years, and to identify factors associated with unfavorable outcomes. Patients receiving intradetrusor injections of 100 U onabotulinumtoxinA for refractory OAB between 2011 and 2021 were retrospectively reviewed. Urodynamic parameters, underlying comorbidities, subjective success, and unfavorable outcomes were assessed. A total of 192 patients were included, and 65 of them were classified into the elderly group. For the elderly group, 60.0% experienced subjective dryness, and 84.6% remained subjective success at 6 months after the injections. The prevalence rates of common unfavorable outcomes, including urinary tract infections, large post-void residual urine volume, and urinary retention, were 9.2%, 27.7%, and 12.3%, respectively. Multivariate analysis revealed that female, baseline urodynamic parameters, and diabetes mellitus were associated with unfavorable outcomes in the elderly group. Intravesical BoNT-A injections provide comparable therapeutic efficacy and safety concerns in elderly patients with refractory OAB. A thorough consultation for treatment benefits and possible adverse events is mandatory before the procedure.

## 1. Introduction

Overactive bladder (OAB) is a syndrome defined by the International Continence Society and is characterized by urinary urgency, with or without urgency urinary incontinence, and usually accompanied by frequency and nocturia [1,2]. Large population-based surveys revealed that the prevalence of OAB increases with age, and is slightly higher for elderly males over females [3,4]. Many studies have described the negative influence of OAB on health-related quality of life, including anxiety, depression, sleep disorder, social withdrawal, and sexual life impairment [5,6,7,8,9,10]. Likewise, urinary incontinence is known to negatively affect the quality of life in the elderly population [11] and is also responsible for low self-esteem and depression [12]. To treat the bothersome storage symptoms of OAB, antimuscarinic agents have been developed to inhibit spontaneous detrusor smooth muscle contractions and reduce afferent signals during bladder filling [13]. The therapeutic effect of antimuscarinic agents has been proven; however, insufficient symptom relief and concomitant adverse events cause poor medication persistence and adherence [14]. Solifenacin and fesoterodine have been shown to have limited impact on cognitive function and few central nervous system adverse events for patients ≥65 years after short-term exposure [15,16,17]. Even though, uncertainty regarding cognitive decline after long-term cumulative anticholinergic exposure still limits their use in the elderly population [18,19]. On the other hand, β3-adrenoceptor agonists facilitate relaxation of the detrusor muscle during bladder filling. Both mirabegron and vibegron have been confirmed to be effective and well-tolerated in the elderly population [20,21,22,23,24,25]. However, most participants in the clinical trials were relatively healthy and did not have uncontrolled cardiovascular diseases. The long-term application of these pharmacological agents in the elderly population remains questionable.

The efficacy of intravesical injection of botulinum toxin type A (BoNT-A) has been established for patients with OAB who have an insufficient response to first-line pharmacological agents [26,27]. However, most of these published data did not focus on the elderly population, and only a handful of studies included a population with a mean age of ≥65 years old [28,29,30,31,32,33,34,35]. In addition, 75 years of age has been proposed as a new cutoff value to redefine the elderly because of the global extension of life expectancy [36]. Exploring the therapeutic outcomes and adverse events associated with BoNT-A injections in this vulnerable population is urgently necessary [37]. The commonly reported adverse events after BoNT-A injection include large post-void residual urine volume (PVR), urinary retention, and urinary tract infection (UTI) [38]. However, factors that can help identify patients at risk of these unfavorable outcomes are still limited, especially in the elderly population. Therefore, the primary aim of our study is to retrospectively evaluate the therapeutic efficacy of intravesical BoNT-A injection for refractory OAB, and the secondary aim is to investigate the factors associated with unfavorable outcomes in an elderly population aged ≥75 years.

## 2. Results

In total, 192 patients received intravesical BoNT-A injections for refractory OAB symptoms during the study period. During the administration of injections, 65 (33.9%) patients were classified into the elderly group (≥75 years old), and the remaining 127 (66.1%) patients were classified into the young group. For the young and elderly groups, the mean age was 58.8 ± 11.9 and 82.0 ± 4.6 years old, respectively. A higher percentage of males was found in the elderly group compared to the young group (75.5% and 34.6%, respectively). More comorbidities were found in the elderly group, including hypertension, diabetes mellitus (DM), dementia, coronary artery disease, and chronic kidney disease. The multichannel urodynamic parameters for baseline bladder function prior to BoNT-A injection are listed in Table 1. For the filling phase parameters, the elderly bladders are more sensitive to filling, and have a smaller cystometric bladder capacity (CBC) compared to those of the young. For the voiding phase parameters, a higher detrusor pressure at the maximal flow rate (PdetQmax), a slower maximum flow rate (Qmax), and a smaller voided volume (VV) were found for the elderly group compared to the young group.

Primary outcomes after intravesical BoNT-A injections are shown in Table 2. At 6 months after the injection, 77.2% and 84.6% of patients in the young and elderly group remained subjective success, respectively. The subjective success rate was comparable in both groups at 3, 6, and 12 months after the injections. Additionally, more than 60% of patients in both groups experienced a certain period of subjective dryness without any urge incontinence. Compared to the baseline uroflowmetry parameters, the CBC and PVR were significantly increased, and the voiding efficiency was significantly decreased in both groups three months postoperatively. In addition, the postoperative CBC and VV were smaller, and Qmax was slower (265.8 ± 126.0 vs. 332.5 ± 158.1 mL, *p* = 0.010; 156.9 ± 106.1 vs. 220.4 ± 139.6 mL, *p* = 0.007; and 11.0 ± 7.3 vs. 15.5 ± 10.7 mL/s, *p* = 0.007, respectively) in the elderly group than in the young group. The prevalence of unfavorable outcomes such as a large PVR, urinary retention, and UTI did not vary between groups. For the young and elderly group, 29 (22.8%) and 18 (27.7%) patients were found to have large PVR, and 11 (8.7%) and 8 (12.3%) patients eventually experienced urinary retention and required catheterization to empty the bladder, respectively. Indwelling Foley catheters were used for all the 11 patients in the elderly group and 4 patients in the young group. Clean intermittent catheterization was used by the other 4 patients in the young group. The catheterization period persisted within one week for 7 patients, between one week to one month for 5 patients, and up to two months for 7 patients. Additionally, 18 (14.2%) and 6 (9.2%) patients in the young and elderly group experienced UTI, respectively.

Table 3 shows the baseline clinical characteristics and multichannel urodynamic parameters of elderly patients with or without postoperative unfavorable outcomes: 6 (9.2%), 18 (27.7%), and 8 (12.3%) patients in the elderly group had postoperative UTI, large PVR, and urinary retention, respectively. For baseline multichannel urodynamic parameters, patients with postoperative UTI tended to have lower bladder compliance (19.7 ± 12.5 vs. 61.0 ± 67.3 mL/cmH_2_O, *p* = 0.014) and a higher PdetQmax (56.2 ± 33.2 vs. 29.0 ± 17.4 cmH_2_O, *p* = 0.013) compared to those without UTI, whereas patients with postoperative large PVR or with urinary retention tended to have higher PdetQmax (40.1 ± 23.4 vs. 28.3 ± 18.6 cmH_2_O, *p* = 0.029; 55.5 ± 27.1 vs. 28.2 ± 17.3 cmH_2_O, *p* = 0.001, respectively) compared to those with normal PVR or without urinary retention. Regarding underlying comorbidities, patients with postoperative UTI had a higher prevalence of dementia, while patients suffering postoperative urinary retention had a greater prevalence of DM and cerebrovascular accidents. 

For the elderly population, multivariate analysis revealed that female, lower baseline bladder compliance, and higher PdetQmax were significantly associated with postoperative UTI. In addition, a higher baseline PdetQmax and a history of DM were associated with urinary retention. However, the association between higher baseline PdetQmax and postoperative large PVR failed to achieve significance (OR: 1.027, *p* = 0.075) after adjusting for age and gender (Table 4).

Forty-three (33.9%) patients in the young group and 14 (21.5%) patients in the elderly group received subsequent injection cycles after the initial BoNT-A effect vanished, whereas the other 135 (70.4%) patients received only one episode of BoNT-A injection. The injection cycles between young and old patient groups are shown in Table 5.

## 3. Discussion

The role of BoNT-A in treating refractory OAB is well established in both sexes [26,27]. However, studies focusing on efficacy and adverse events in the elderly population are limited [38]. In addition, with the extension of life expectancy, “75 years of age and over” is increasingly being used to define the elderly population [36]. Hence, our study defined the elderly population as patients aged 75 years or older. We aimed to determine the therapeutic outcome of intravesical BoNT-A in this population and identify valuable factors associated with adverse events. Our results revealed that although elderly bladders were more sensitive at baseline compared to young bladders, BoNT-A intravesical injection was equally effective for OAB symptom control. In addition, the prevalence of adverse events was equal in both age groups. Female sex, lower bladder compliance, and higher PdetQmax were associated with postoperative UTI, while DM and higher PdetQmax were associated with postoperative urinary retention in the elderly population.

Several possible pathophysiologies have been proposed to explain refractory OAB [39], including urothelial dysfunction with aging [40], undetected bladder outlet obstruction (BOO), chronic bladder ischemia or inflammation [41,42], and central sensitization [43,44]. These conditions are commonly found in the elderly population owing to aging-induced changes from the brain to the bladder itself [45,46,47]. In our study, more than 75% of patients in the elderly group were men, a proportion much higher than that in the young group. This may further emphasize the importance of chronic undetected BOO in bladder remodeling [48]. It is well-documented that the presence of BOO will result in large PVR and could be a risk of urinary retention after the intravesical BoNT-A injection, especially in the elderly [29,30]. Therefore, in our clinical practice, we will investigate patients with refractory OAB by video-urodynamic study to find if there is BOO, and the BoNT-A injection can only be performed in patients without BOO, or if their BOO has been well-treated. In addition, our findings of the preoperative multichannel urodynamic study in these elderly bladders, including increased bladder sensation and reduction in bladder capacity, were consistent with the known changes in the aging bladder [46]. Intravesical BoNT-A injection provides sensory blockade in addition to chemo-denervation of the bladder detrusor muscle [49,50]. This may explain why patients who are refractory to conventional OAB medications can be successfully treated with BoNT-A.

To the best of our knowledge, no case-control study has compared the therapeutic efficacy of BoNT-A between patients aged ≥75 years and those aged <75 years. White et al. [34] reported a case series of 21 refractory OAB patients aged 75 years and older and concluded that BoNT-A injection is efficacious, durable, and has a low incidence of adverse events in the short term. Frailty has been proposed as a negative factor for long-term treatment success, but this study used “age greater than 65 years” as the definition of elderly [29]. Our study demonstrated that the elderly population (≥75 years old) had similar subjective success rates at 3, 6, and 12 months postoperatively compared with the young population. Furthermore, with no between-group differences, >60% of patients in both groups eventually experienced a certain period of subjective dryness without urge incontinence. This highlights that age itself is not a direct factor that affects the bladder response to BoNT-A. Instead, the underlying pathophysiologies that develop during the aging process to induce refractory OAB are key factors in determining therapeutic outcomes.

Considering the direct chemo-denervation effect on the bladder detrusor muscle, PVR elevation and urinary retention are common concerns after intravesical BoNT-A injections [51]. A large PVR is commonly defined as a PVR greater than 150 or 200 mL, and approximately 6–61% of patients with a mean age > 65 years have been reported to experience a large PVR after receiving injections [28,29,30,35]. Miotla et al. reported that female patients with PVR > 200 mL or retention after injections were older than those with PVR < 200 mL [52]. Liao and Kuo proposed that instead of age, frailty was associated with post-injection PVR > 150 mL [29]. In our elderly population (≥75 years old), 18 (27.7%) patients were found to have a large PVR > 200 ml after BoNT-A intravesical injection, and eight (12.3%) patients eventually experienced urinary retention and needed temporary Foley catheter indwelling. There was no difference in the prevalence of a large PVR and urinary retention between the elderly and young populations. Although no valuable factor could be found to be associated with large postoperative PVR in our elderly population, a higher baseline PdetQmax and a history of DM were identified as factors associated with postoperative urinary retention. DM is a well-known factor that induces overactive bladder and affects detrusor contractility during the voiding phase [53]. Wang et al. reported that intravesical BoNT-A successfully managed detrusor overactivity and achieved a similar treatment success rate in both DM and non-DM patients but with a higher risk of large PVR and general weakness in DM patients [54]. In elderly patients with DM, detailed consultation and close follow-up for postoperative PVR are necessary. 

UTI is another common but frustrating unfavorable outcome after intravesical BoNT-A injections [55]. A recent systemic review revealed that the prevalence rate of UTI after intravesical BoNT-A injection for treating OAB is approximately 29.8% [56]. Both storage and emptying dysfunction have been proposed to impact UTI recurrence [57,58]. In our study, we found that female sex, lower bladder compliance, and a higher PdetQmax were associated with postoperative UTI in the elderly population. Lower bladder compliance and higher PdetQmax are common bladder dysfunctions that increase intravesical pressure during both the storage and emptying phases. Increased intravesical pressure is known to cause bladder ischemia, which may predispose the bladder to infection because of a delayed or insufficient immune response [59,60]. 

Although the present study successfully demonstrated the therapeutic outcomes and adverse events of intravesical BoNT-A injections in a population older than most of the published data, some limitations still exist. First, its retrospective design made it possible to involve biases during patient selection, data collection, and statistical analysis. Moreover, we could not further define ‘frailty’ by retrospectively reviewing the medical records. Instead, we believe that using 75 years as the cutoff value would be indeed a reasonable choice. Second, the small sample size in the elderly group limited the statistical power in multivariate logistic regression analyses, and also hindered the subgroup analysis for different sexes. Third, no postoperative multichannel urodynamic data were available to provide detailed bladder storage function after BoNT-A injection. Considering the invasiveness of the test, a simple uroflowmetry with PVR is commonly used to represent postoperative bladder function. Finally, in the long-term follow-up, we found only 29.7% of refractory OAB patients received subsequent BoNT-A injection in our hospital. This result indicates that the patients might not be satisfied with the unfavorable treatment outcome after the first BoNT-A injection and would choose medical therapy for their bothersome OAB symptoms. However, understanding the treatment effect of BoNT-A on the sensory blockade in the elderly population remains limited. Prospective case-control studies are necessary to evaluate treatment outcomes and outcome predictors in this population in detail.

## 4. Conclusions

Intravesical BoNT-A injections provided equally effective and durable therapeutic outcomes in both young and elderly patients (≥75 years old) with refractory OAB. The prevalence rates of common unfavorable outcomes were equal between the two age groups. For elderly patients receiving intravesical BoNT-A injection, female, lower bladder compliance, and higher PdetQmax were associated with postoperative UTI, whereas a history of DM and higher PdetQmax were associated with urinary retention postoperatively. A thorough consultation for possible benefits and adverse events is mandatory, especially in elderly patients with certain risk factors.

## 5. Materials and Methods

We retrospectively reviewed patients with idiopathic OAB symptoms refractory to conventional medications who received intravesical injections of BoNT-A for the first time at a tertiary medical center in eastern Taiwan. All patients had persistent urgency urinary incontinence even with antimuscarinics, β3-adrenoceptor agonists, or a combination of both for more than three months. A multichannel urodynamic study, including cystometry and a pressure flow study, was performed preoperatively in accordance with the International Continence Society’s good urodynamic practice recommendations [61] to confirm the presence of detrusor overactivity. All patients have been proven to be non-BOO by the video-urodynamic study before receiving the intravesical BoNT-A injections. Patients with underlying neurological factors that may cause neurogenic detrusor overactivity, or intrinsic sphincter deficiency were excluded from this study. Patients who were ≥75 years old while receiving the injections were classified into the elderly group, whereas the others belonged to the young group. 

Baseline lower urinary tract function was assessed for each patient using uroflowmetry, PVR, and a multichannel urodynamic study before BoNT-A injection. Parameters including the VV, Qmax, CBC, and voiding efficiency were collected from the uroflowmetry. CBC was defined as the sum of VV and PVR, and voiding efficiency was defined as VV divided by CBC. For the multichannel urodynamic study, bladder sensations, compliance, and the presence of detrusor overactivity were recorded as the filling phase parameters, whereas PdetQmax, Qmax, VV, CBC, and PVR were recorded as the voiding phase parameters. BOO was defined as BOO index >40 for men [62], and as PdetQmax > 35 cmH_2_O for women [63]. Bladder sensations were further classified as the first sensation of filling, full sensation, and urge sensation, according to the patients’ reports. 

All patients were hospitalized and received intravesical injections of 100 units of Botox^®^ (Allergan, Irvine, CA, USA), which is the standard dosage used to treat refractory OAB [26], under general anesthesia in the operating room. The injection method has been described previously [26]. Briefly, 10 mL normal saline was used to dilute each Botox vial. The injection needle was inserted into the posterior and lateral bladder walls under the guidance of rigid cystoscopy, and a total of 20 evenly distributed intradetrusor injections (0.5 mL for each injection) were performed while sparing the trigone area. A 14 Fr. urethral Foley catheter was placed and remained in place for one day after the Botox injection. Objective outcomes were assessed three months after the injections using uroflowmetry and PVR. Subjective treatment success and improvement of urge incontinence were reviewed according to the medical records at the out-patient department during serial follow-ups. As improvement of urinary incontinence and difficult urination might coexist after intravesical BoNT-A injection, patients might consider that they had unsuccessful treatment if they had severe difficulty in urination even though urinary incontinence had improved. Therefore, a subjective success was defined by having a Global Response Assessment (scoring from −3 to +3, indicating markedly worse to markedly improved after the treatment [64]) of +2 or +3. Underlying comorbidities and postoperative unfavorable outcomes, including a large PVR (defined as PVR >200 mL during the follow-up period), urinary retention, and UTI, were also collected from the patients’ medical records. All patients were followed up regularly at the out-patient clinic with or without OAB medication, and repeat BoNT-A injections were performed if patients had recurrence of OAB symptoms and requested for injection, otherwise they were continuously treated with oral medications.

Statistical analyses were performed using SPSS Statistics for Windows, Version 17.0. Chicago: SPSS Inc. Continuous and categorical variables are expressed as mean ± standard deviation and as numbers and percentages, respectively. Statistical comparisons between groups were performed using the Mann–Whitney U test for continuous variables and the chi-square test for categorical variables. Fisher’s exact test was applied when > 20% of the expected frequencies were less than five. Comparisons between baseline and follow-up within-group differences were performed using the Wilcoxon signed-rank test. Age, gender, and variables demonstrating significant differences between patients with or without each unfavorable outcome were further analyzed with multivariate logistic regression analyses to identify factors associated with postoperative unfavorable outcomes in the elderly group. All statistical assessments were considered significant when the two-sided *p*-value was <0.05.

## Figures and Tables

**Table 1:** Patient demographics and baseline multichannel urodynamic parameters.

	Young Group (<75) Years	Elderly Group (≥75 Years)	
	Mean ± SD or No. (%)	Mean ± SD or No. (%)	*p*-Value
Number of patients	127	65	
Age (years)	58.8 ± 11.9	82.0 ± 4.6	
Gender (male)	44 (34.6)	51 (75.5)	<0.001
Baseline multichannel urodynamic parameters			
FSF (mL)	110.4 ± 64.4	96.3 ± 66.4	0.051
FS (mL)	171.5 ± 102.1	135.6 ± 87.3	0.010
US (mL)	201.7 ± 117.1	152.4 ± 100.8	0.003
CBC (mL)	272.6 ± 161.1	184.7 ± 109.7	<0.001
Compliance (mL/cmH_2_O)	66.4 ± 76.8	57.2 ± 65.3	0.437
PdetQmax (cmH_2_O)	23.7 ± 16.6	31.5 ± 20.6	0.003
Qmax (mL/s)	12.7 ± 7.8	7.8 ± 4.5	<0.001
VV (mL)	230.3 ± 142.4	155.7 ± 103.4	<0.001
PVR (mL)	42.2 ± 100.8	29.3 ± 50.6	0.109
Comorbidities			
Hypertension	66 (52.0)	46 (70.8)	0.012
DM	24 (18.9)	25 (38.5)	0.003
CVA	16 (12.6)	13 (20.0)	0.175
Dementia	5 (3.9)	10 (15.4)	0.005
CAD	5 (3.9)	13 (20.0)	<0.001
CHF	3 (2.4)	2 (3.1)	1.000
CKD	2 (1.6)	5 (7.7)	0.045

CAD: coronary artery disease; CBC: cystometric bladder capacity; CHF: congestive heart failure; CKD: chronic kidney disease; CVA: cerebrovascular accident; DM: diabetes mellitus; FS: full sensation; FSF: first sensation of filling; No.: number; PdetQmax: detrusor pressure at the maximal flow rate; PVR: post-void residual urine volume; Qmax: maximum flow rate; SD: standard deviation; US: urge sensation; VV: voided volume.

**Table 2:** Primary outcomes and unfavorable outcomes after intravesical BoNT-A injection.

	Young Group (<75 Years) (*n* = 127)	Elderly Group (≥75 Years) (*n* = 65)	*p*-Value
	Mean ± SD or No. (%)	Mean ± SD or No. (%)	
Subjective success (No.)			
At 3 months	124 (97.6%)	63 (96.9%)	1.000
At 6 months	98 (77.2%)	55 (84.6%)	0.225
At 12 months	31 (24.4%)	31 (32.3%)	0.244
Subjective dryness (No.)	85 (66.9%)	39 (60.0%)	0.342
Uroflowmetry			
Qmax (mL/s)			
Baseline	17.0 ± 13.3	11.3 ± 7.4	0.001
3 months	15.5 ± 10.7	11.0 ± 7.3	0.004
VV (mL)			
Baseline	211.8 ± 149.6	138.6 ± 84.7	0.002
3 months	220.4 ± 139.6	156.9 ± 106.1	0.007
PVR (mL)			
Baseline	48.7 ± 85.0	38.5 ± 48.7	0.876
3 months	144.4 ± 114.9 *	149.3 ± 117.3 *	0.757
CBC (mL)			
Baseline	260.6 ± 164.8	177.1 ± 99.1	0.001
3 months	332.5 ± 158.1 *	265.8 ± 126.0 *	0.010
VE (%)			
Baseline	81.8 ± 21.6	79.8 ± 21.0	0.292
3 months	65.2 ± 71.8 *	57.4 ± 27.3 *	0.106
Unfavorable outcomes			
Large PVR (> 200 mL) (No.)	29 (22.8%)	18 (27.7%)	0.459
Urinary retention (No.)	11 (8.7%)	8 (12.3%)	0.423
UTI (No.)	18 (14.2%)	6 (9.2%)	0.327

CBC: cystometric bladder capacity; No.: number; PVR: post-void residual urine volume; Qmax: maximum flow rate; SD: standard deviation; UTI: urinary tract infection; VE: voiding efficiency; VV: voided volume. * Wilcoxon signed-rank test *p* < 0.001 at 3 months compared to baseline.

**Table 3:** Baseline clinical characteristics and multichannel urodynamic parameters in elderly population with or without each unfavorable outcome.

Unfavorable Outcomes	UTI			Large PVR			Urinary Retention		
	No	Yes	*p*-Value	≤200 mL	>200 mL	*p*-Value	No	Yes	*p* Value
Number of patients	59	6		47	18		57	8	
Age (years)	81.9 ± 4.6	82.8 ± 5.5	0.650	81.4 ± 4.5	83.4 ± 4.8	0.106	81.7 ± 4.6	84.1 ± 4.5	0.176
Gender (male)	48 (81.4)	3 (50.0)	0.108	36 (76.6)	15 (83.3)	0.740	44 (77.2)	7 (87.5)	0.675
Baseline multichannel urodynamic parameters									
FSF (mL)	98.1 ± 68.9	78.8 ± 29.5	0.903	94.6 ± 66.6	100.8 ± 67.6	0.747	95.7 ± 67.8	100.4 ± 59.6	0.660
FS (mL)	137.1 ± 89.7	120.8 ± 62.1	0.903	133.7 ± 86.2	140.3 ± 92.6	0.889	135.1 ± 86.5	138.6 ± 98.9	0.944
US (mL)	154.5 ± 103.9	131.8 ± 66.8	0.834	150.0 ± 97.4	158.7 ± 112.1	1.000	152.4 ± 100.5	152.8 ± 110.2	0.992
CBC (mL)	189.8 ± 111.1	135.0 ± 88.2	0.223	179.7 ± 96.7	197.7 ± 140.6	0.953	188.3 ± 109.7	159.3 ± 113.5	0.442
Compliance (mL/cmH_2_O)	61.0 ± 67.3	19.7 ± 12.5	0.014	53.9 ± 63.1	65.7 ± 71.8	0.639	59.0 ± 68.2	43.9 ± 39.5	0.472
PdetQmax (cmH_2_O)	29.0 ± 17.4	56.2 ± 33.2	0.013	28.3 ± 18.6	40.1 ± 23.4	0.029	28.2 ± 17.3	55.5 ± 27.1	0.001
Qmax (mL/s)	7.9 ± 4.4	6.5 ± 5.7	0.485	7.8 ± 4.5	7.6 ± 4.6	0.860	7.9 ± 4.5	6.6 ± 4.5	0.496
VV (mL)	162.5 ± 102.4	85.0 ± 92.7	0.048	154.7 ± 93.6	157.2 ± 128.5	0.671	159.6 ± 99.8	125.5 ± 130.0	0.235
PVR (mL)	27.2 ± 47.9	50.0 ± 75.4	0.214	25.0 ± 47.0	40.6 ± 59.1	0.332	28.7 ± 48.2	33.8 ± 69.5	0.550
Comorbidities									
Hypertension	42 (71.2%)	4 (66.7%)	1.000	33 (70.2)	13 (72.2)	0.873	39 (68.4)	7 (87.5)	0.420
DM	24 (40.7%)	1 (16.7%)	0.393	15 (31.9)	10 (55.6)	0.080	19 (33.3)	6 (75.0)	0.047
CVA	11 (18.6%)	2 (33.3%)	0.591	7 (14.9)	6 (33.3)	0.162	9 (15.8)	4 (50.0)	0.044
Dementia	7 (11.9%)	3 (50.0%)	0.042	7 (14.9)	3 (16.7)	1.000	7 (12.3)	3 (37.5)	0.098
CAD	11 (18.6%)	2 (33.3%)	0.591	9 (19.1)	4 (22.2)	0.743	12 (21.1)	1 (12.5)	1.000
CHF	2 (3.4%)	0 (0.0%)	1.000	2 (4.3)	0 (0.0)	1.000	2 (3.5)	0 (0.0)	1.000
CKD	4 (6.8%)	1 (16.7%)	0.394	4 (8.5)	1 (5.6)	1.000	4 (7.0)	1 (12.5)	0.493

CAD: coronary artery disease; CBC: cystometric bladder capacity; CHF: congestive heart failure; CKD: chronic kidney disease; CVA: cerebrovascular accident; DM: diabetes mellitus; FS: full sensation; FSF: first sensation of filling; No.: number; PdetQmax: detrusor pressure at the maximal flow rate; PVR: post-void residual urine volume; Qmax: maximum flow rate; SD: standard deviation; US: urge sensation; UTI: urinary tract infection; VV: voided volume. Data expressed by mean ± standard deviation or number (%).

**Table 4:** Univariate and multivariate logistic regression to identify factors associated with each unfavorable outcome in elderly population.

	Postoperative UTI (*n* = 6)			Large PVR > 200 mL (*n* = 18)			Urinary Retention (*n* = 8)		
	OR (95% CI)	Adjusted OR (95% CI)	*p*-Value **	OR (95% CI)	Adjusted OR (95% CI)	*p*-Value **	OR (95% CI)	Adjusted OR (95% CI)	*p*-Value **
Age	1.044 (0.873–1.249)	1.344 (0.892–2.026)	0.157	1.101 (0.975–1.242)	1.099 (0.960–1.257)	0.170	1.120 (0.953–1.315)	1.245 (0.942–1.646)	0.124
Gender (male)	0.229 (0.041–1.292)	0.000 (0.000–0.400)	0.029	1.528 (0.372–6.268)	0.803 (0.167–3.866)	0.785	2.068 (0.233–18.382)	0.309 (0.018–5.255)	0.416
Compliance	0.951 (0.904–1.000)	0.903 (0.820–0.995)	0.040	1.003 (0.995–1.010)			0.994 (0.976–1.013)		
PdetQmax	1.048 (1.009–1.089) *	1.214 (1.007–1.464)	0.042	1.028 (0.999–1.057)	1.027 (0.997–1.058)	0.075	1.058 (1.013–1.104) *	1.077 (1.012–1.145)	0.019
DM	0.292 (0.032–2.656)			2.667 (0.876–8.122)			6.000 (1.104–32.595) *	29.042 (1.114–756.870)	0.043
CVA	2.182 (0.354–13.458)			2.857 (0.805–10.143)			5.333 (1.123–25.331) *	4.683 (0.587–37.359)	0.145
Dementia	7.429 (1.247–44.239) *	0.026 (0.000–3.144)	0.136	1.143 (0.261–5.005)			4.286 (0.835–21.991)		

CI: Confidence interval; CVA: cerebrovascular accident; DM: diabetes mellitus; OR: odds ratio; PdetQmax: detrusor pressure at the maximal flow rate; PVR: post-void residual urine; UTI: urinary tract infection. * Univariate logistic regression *p* < 0.05. ** Multivariate Binary logistic regression.

**Table 5:** The subsequent BoNT-A injection cycles during the follow-up period after the first time BoNT-A injection for young and elderly patients with refractory overactive bladder.

Subsequent Injection Cycle(s)	0	1	2	3	4	5	6	7
Young patients (n)	84	22	10	4	2	2	2	1
Elderly patients (n)	51	10	3	1	0	0	0	0

## Data Availability

Data are available on request to the corresponding author.
